# N-cadherin mimetic hydrogels drive superior regenerative and paracrine responses in 3D cultures of adipose-derived mesenchymal stem cells

**DOI:** 10.55730/1300-0152.2738

**Published:** 2025-02-03

**Authors:** Demet KAÇAROĞLU, Alper Murat ULAŞLI, Aybüke Didenur SAK, Seher YAYLACI

**Affiliations:** 1 Department of Medical Biology, Faculty of Medicine, Lokman Hekim University, Ankara, Turkiye; 2 Interdisciplinary Stem Cell and Regenerative Medicine Department, Stem Cell Institute, Ankara University, Ankara, Turkiye; 3 Department of Biology, Faculty of Science, Middle East Technical University, Ankara, Turkiye

**Keywords:** Mesenchymal stem cell, regenerative medicine, N-cadherin, hydrogels, 3D culture, biomimetic material

## Abstract

**Background:**

Cadherin-based biomaterials play a pivotal role in influencing the fate of mesenchymal stem cells (MSC). Enhancing the adhesion of adipose tissue-derived MSCs has been shown to augment their paracrine effects while N-cadherin biomaterials have been suggested to regulate the paracrine effects of MSCs via specific growth factors although the precise mechanisms underlying this regulation remain insufficiently understood. This study aims to compare the effects of a 3D N-cadherin mimetic environment on cell viability, apoptosis, extracellular matrix regulation, and growth factor expression with those observed in traditional 2D and 3D spheroid cultures. Additionally, the study seeks to evaluate the effects of conditioned media derived from the N-cadherin mimetic environment on the viability and migration of endothelial cells.

**Materials and methods:**

Peptide hydrogels, including HAVDI and SCRAM, were used as N-cadherin mimetics at a concentration of 1 mM, and four experimental groups were established: 2D classical culture, 3D spheroid culture, 3D HAVDI, and 3D SCRAM. Cell viability was assessed using the MTT assay, while gene expression analysis (*BCL-XL*, *BCL-2*, *BAX*, *MMP-9*, *TIMP1*, *MMP-2*, *PLAU*, *HGF*, *FGF*, and *VEGFR2*) was performed via qRT-PCR. Secretion levels of growth factors (PDGF-BB, FGF-2, and VEGF-A) were quantified using ELISA. The effects of conditioned media on the proliferation and migration of human umbilical vein endothelial cells were evaluated through MTT assays, calcein staining, and wound healing assays.

**Results:**

In the 3D HAVDI group, where MSCs were cultured in an N-cadherin mimetic peptide environment, cell viability increased, and apoptosis decreased. Moreover, this environment upregulated genes associated with tissue remodeling and increased the expression and secretion of growth factors, compared to the classical 2D culture. Additionally, treatment with conditioned media at 1:2 and 1:5 dilutions significantly improved the viability and migration potential of endothelial cells.

**Conclusion:**

The N-cadherin mimetic peptide hydrogel represents a more effective culturing strategy than traditional 2D for enhancing the paracrine and regenerative properties of MSCs.

## 1. Introduction

High-regenerative mesenchymal stem cells (MSCs) have immunomodulatory properties, making them useful for various medical applications (Pountos and Giannoudis, 2005). They can develop into mesodermal cells like bone, cartilage, muscle, fat, and tendon (Squillaro et al., 2016). MSCs affect nearby cells by secreting cytokines, chemokines, and exosomes, or through direct contact (Zhou et al., 2021). Cell viability and therapeutic potency are dependent on in vitro signaling, as MSCs respond to both cell-cell and cell-environment signals (Han et al., 2019).

Biocompatible synthetic or natural biomaterials can modulate their biological responses, and peptide-based synthetic materials are used in cell culture investigations (Place et al., 2009; Hussey et al., 2019). N-cadherins, which connect to two neighbors extracellularly and link to actins cytoplasmically via catenins, regulate embryo development, tissue fibrosis, and cancer progression (Ozawa et al., 1989). They also affect cell proliferation, differentiation, and apoptosis (Brusés, 2006). MSC behavior is affected by N-cadherin mimetic peptides, which resemble the native ECM (Li et al., 2017).

Physiological mechanisms include apoptosis, which can be prevented by caspase activation and PARP inactivation (Jourdan et al., 2009). In vitro studies have linked N-cadherin to adhesion-dependent apoptosis prevention in nonneuronal cells (Parnaud et al., 2011; Lelièvre et al., 2012). In embryonal cancer cells, N-cadherin-Fc fusion protein increased colony formation and proliferation (Yue et al., 2010). Polylactic-co-glycolic acid microparticles with E-cadherin fusion protein incorporated into MSCs increased cell proliferation, AKT-ERK1/2 signaling, and EGF-HGF secretion (Zhang et al., 2016a, Zhang et al., 2016b). Cadherin-mimetic peptides are expected to prevent apoptosis, which is essential for ADMSC survival.

Fibrosis, caused by excessive extracellular matrix protein accumulation, can lead to tissue scarring and dysfunction (Agarwal, 2014). HAVDI peptide fragments modify hydrogel stiffness-induced signaling and may alter tissue remodeling genes (Ferrell et al., 2022). ADMSCs secrete proteins that regulate angiogenesis, reepithelialization, cell migration/differentiation/survival/death, and immune modulation, affecting wound healing (Qin et al., 2020, Almalki, 2022). Investigating the effects of environmental VEGF protein on endothelial cells is crucial for wound healing.

This study aimed to compare the effects of hydrogels prepared in a 1 mM N-cadherin mimetic environment on ADMSCs cell viability, apoptosis, ECM remodeling, growth factor secretion, and endothelial cell activation in both 2D classical and 3D spheroid cultures.

## 2. Material and methods

### 2.1. Peptide synthesis and self-assembled peptide hydrogel characterization

All required amino acids, 2-(1H-benzotriazol-1-yl)-1,1,3,3-tetramethyluronium hexafluorophosphate (HBTU), Rink amide 4-methylbenzhydrylamine (MBHA) resin (ranging from 0.3 to 0.6 mmol/g, 100–200 mesh size), and Lauric acid were sourced from Chem-Impex International, based in Wood Dale, IL, USA. Additionally, piperidine, trifluoroacetic acid (TFA), and isopropyl silane (TIS) were acquired from Acros (Beijing, China). For water purification, a double distillation process was implemented using a Millipore simplicity 185 system (Darmstadt, Germany), yielding water of 18.1 MΩ purity. Peptides were manually synthesized on Rink amide (MBHA resin using fluorenylmethoxycarbonyl (Fmoc)-based solid-phase peptide synthesis (SPPS) under ambient temperature and standard atmospheric conditions. The resin was swollen in dimethylformamide (DMF) for at least 30 min, and amino acid coupling was performed using three equivalents of Fmoc-protected L-amino acids and HBTU, along with six equivalents of N,N-diisopropylethylamine (DIPEA). The resin was treated twice with 20% piperidine in DMF to remove Fmoc protective groups. The Kaiser test was performed to verify peptide coupling and Fmoc elimination. After peptide chain completion, the resin was washed, dried, and cleaved using TFA, TIS, and water. The peptides were filtered, precipitated with cold diethyl ether, and centrifuged. After air-drying, the peptides were purified by reversed-phase high-performance liquid chromatography (HPLC) and then lyophilized into pure powders. (Eren Cimenci et al., 2019). Peptide analysis was performed on a Dionex UltiMate 3000 HPLC system with a Gemini-NX C18 column. The structural composition of the peptides was validated using an Agilent Technologies 6530 Accurate-Mass Q-TOF instrument in positive-ion mode (Eren Cimenci et al., 2019).

### 2.2. Adipose-derived mesenchymal stem cell culture and immunophenotyping

Human ADMSCs (PCS-500-011 TM) were obtained by ATCC (Wesel, Germany). Human ADMSCs were grown in low-glucose DMEM (Capricorn, Germany) media with 20% fetal bovine serum (Capricorn, Germany), 1% L-glutamine (Capricorn, Germany), and 1% penicillin/streptomycin (BIOIND, Israel) (Bunnell et al., 2008). Cultures were incubated at 37 °C with 5% CO_2_ and 100% humidity for 70%–80% confluency. To determine the expression level of three different surface markers used for immunophenotyping (CD73, CD90, and CD105), 1,000,000 cells were analyzed by flow cytometry (Bourin et al., 2013). The cells were then stained using the MSC marker verification kit (R&D Systems, Catalog #FMC020). The MSC marker kit was used to stain 50,000 ADMSCs with both positive and negative control antibodies. The cell pellet was resuspended in 100 μL of staining solution for flow cytometry analysis. Cellquest Pro software was used to view fluorescence results as histograms and calculate expression percentages relative to isotype-matched controls (Kaçaroğlu and Yaylacı, 2024).

### 2.3. 2D covered and 3D hydrogel N-cadherin mimetic culture environment design

The study investigated the effects of an N-cadherin biomimetic milieu on ADMSCs in three dimensions using hydrogels. Peptide amphiphiles HAVDI-PA, SCRAM-PA, and K-PA were produced at 1 mM for 2D and 3D hydrogel culturing. A 2D culture environment was created on a 96-well plate with an uncoated tissue culture plate (2D TCP) as the control. Cell viability was evaluated in 2D by administering a 50 μL mixture of 1 mM HAVDI-PA and 1 mM SCRAM-PA with K-PA to the well bottoms (Eren Cimenci et al., 2019). The coated plates were dried for 24 h, and ADMSCs were seeded on uncoated and coated surfaces for viability investigation. In a 24-well plate, three experimental groups were created for the 3D culture model: spheroid, HAVDI, and SCRAM. The hydrogels were prepared, and 80 μL of medium containing 150,000 cells was added to each well. The cells and supernatant from each batch of 900,000 cells planted in six wells were utilized for qPCR and ELISA tests. The conditioned media from the 2D TCP, 3D spheroid, 3D HAVDI, and 3D SCRAM groups were diluted with new complete medium at varied ratios and used in human endothelial cell assays. Viability analyses were performed with six independent experimental groups. Migration analysis, PCR, and ELISA experiments were performed with three independent experimental groups.

### 2.4. Cell viability analysis

The study used the MTT assay to evaluate the effects of HAVDI and SCRAM peptide amphiphile on ADMSCs in 2D cell culture. The viability percentages represent the metabolic activity of viable cells as measured by the MTT assay and are not indicative of cell proliferation or growth rate. The 2D TCP control did not exhibit cytotoxic effects on cells. The cells were seeded into 96-well plates and incubated at 37 °C with 5% CO_2_ for 24 h. Subsequently, 10 μL of MTT solution (Biotium, California, USA) and 90 μL of DMEM media were added to each well at 24, 48, and 72 h. The plates were incubated in the dark for 4 h to create formazan crystals, which were then dissolved in dimethyl sulfoxide and mixed by pipetting. The absorbance of the dissolved formazan solution was measured at 570 nm using a BioTek Synergy H1 microplate reader (Kaçaroğlu and Yaylacı, 2024). The test assessed the biocompatibility of HAVDI and SCRAM peptides with ADMSCs.

### 2.5. Gene expression analysis

The N-Cadherin biomimetic platform was tested for its effect on gene expression in ADMSCs by isolating RNA from 900,000 cells per experimental group, with each group consisting of 6 wells. Plates were incubated for 48 h at 37 °C with 5% CO_2_. Total RNA was extracted using Trizol (SERVA, Germany) according to the manufacturer’s instructions. A LightCycler 96 instrument was used for qRT-PCR using the A.B.T. 2X qPCR SYBR-Green Master Mix kit (ATLAS Biotechnology, Türkiye) (Kacaroglu et al., 2024). Primers were synthesized by Thorvacs Biotechnology (Ankara, Türkiye) using Primer3web version 4.1.0. The following were analyzed: glyceraldehyde-3-phosphate dehydrogenase (*GAPDH*), matrix metalloproteinases 2 and 9 (*MMP-2* and *MMP-9*), B-cell lymphoma 2 protein (*BCL-2*), *BCL-XL*, *BAX*, plasminogen activator urokinase (*PLAU*), *VEGFR2*, and tissue inhibitor of metalloprotein (*TIMP1*). The sequences of the primers are shown in Table.

### 2.6. Quantification of growth factors secreted from ADMSCs

This phase of the experiment measured ADMSC growth factor concentrations using ELISA. Human FGF-2, VEGF-A, and PDGF-BB ELISA kits were supplied by ELK Biotechnology. The experimental groups (2D TCP, 3D Spheroid, 3D HAVDI, and 3D SCRAM) were cultivated in 24-well plates, with 900,000 cells per well. Plates were incubated for 48 h at 37 °C with 5% CO_2_. After incubation, the cells from the four experimental groups were placed in tubes and centrifuged for 5 min at 37 °C and 1500 × *g*. A ten-fold dilution was followed by meticulous collection and analysis of the supernatant. The ELISA kits were performed according to the manufacturer’s protocol, with three controls per group. Standards and samples were prepared concurrently, and OD values were determined at 450 nm using a microplate reader (BioTek Synergy H1, Turkiye) (Kaçaroğlu and Yaylacı, 2024). The pg/ml concentrations of each growth factor in the samples were estimated from the OD values and standard curves.

### 2.7. Assessment of proliferation and migration of conditioned media on endothelial cells

The N-cadherin biomimetic milieu was tested on the proliferation and migration of human umbilical vein endothelial cells (HUVECs) using conditioned media from the 3D Spheroid, 3D HAVDI, and 3D SCRAM groups. HUVEC cells were grown in DMEM/F-12 media with 10% FBS and 1% penicillin/streptomycin at 37 °C, 5% CO_2_, and 100% humidity. Cell viability was assessed by adding 2 μM calcein-acetoxymethyl ester (Santa Cruz Biotechnology, Inc., Dallas, TX, USA) to the conditioned medium group and incubating at 37 °C for 30 min in the dark. Leica Microsystems (Wetzlar, Germany) DM IL fluorescent inverted microscopes were used to picture live cells. The migration assay involved scratching the bottom of 24-well plates when the cells reached 90% confluence. The migration assay involved scratching the bottom of 24-well plates with a pipette tip when the cells reached 90% confluence. The cell-free area of each group was determined as a percentage using ImageJ, and motility was quantified by the percent reduction in the wound cell-free area relative to control cells (Kaçaroğlu et al., 2023).

### 2.8. Statistical analysis

Graphs and statistical analysis were performed in GraphPad Prism 8.1 (GraphPad, San Diego, CA, USA), using mean and standard deviation. Nonparametric two-way ANOVA tests and Tukey’s post hoc tests were used for all statistical analyses and modifications. ADMSCs grown in 2D TCP without treatment were controls. Statistical significance was indicated by an asterisk (*) at p < 0.05. The following symbols were used to denote significance: *p < 0.05, **p < 0.01, ***p < 0.001, ****p < 0.0001.

## 3. Results

### 3.1. Synthesis and characterization of peptides

**Figure 1:**
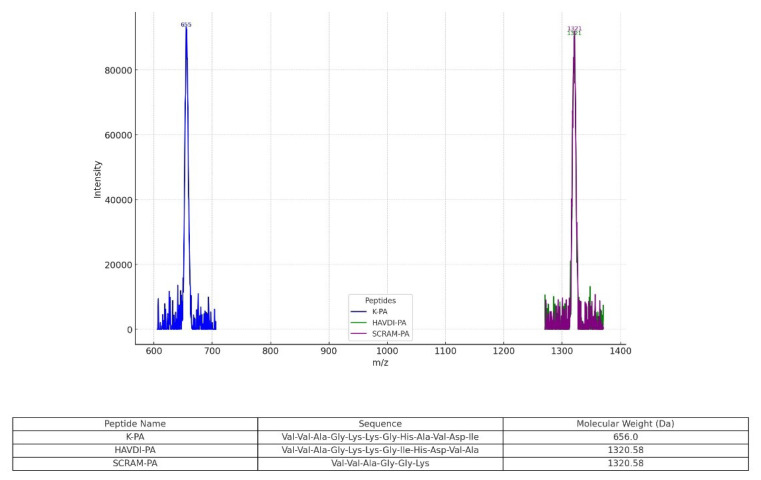
The LC-MS spectra and a table summarizing the peptide sequences, names, and estimated molecular weights for K-PA, HAVDI-PA, and SCRAM-PA

K-PA, HAVDI-PA, and SCRAM-PA were synthesized on Rink amide MBHA resin using Fmoc-based solid-phase peptide synthesis (SPPS) and purified using reversed-phase HPLC. LC-MS analysis, conducted with an Agilent Technologies (Santa Clara, CA, USA) 6530 Accurate-Mass Q-TOF in positive-ion mode, validated the identification and purity of the synthesized peptides. The molecular weights of the peptides were determined and confirmed using LC-MS analysis, which demonstrated peaks corresponding to their calculated molecular weights, as summarized in Figure 1. HAVDI-PA and SCRAM-PA, which have the same amino acid composition but different sequences, showed overlapping peaks at 1320.58, indicating equal molecular weights. The peptide sequences, names, and predicted molecular weights are listed in Figure 1, confirming their effective synthesis and precise mass determination.

### 3.2. Characterization of ADMSCs

Typical fibroblast-like ADMSCs were grown (Figure S a). Stained scatter plots and histograms from flow cytometry analysis are displayed in (Figure S b). The analysis contained 45,686 labeled cells with a gate range of 91.4%. Mesenchymal stem cells displayed 99.7% CD73, 99.91% CD90, 98.71% CD44, and 99.82% CD105 within this gate (Figure S b).

### 3.3. N-cadherin biomimetic microenvironment supports viability and regulates apoptotic gene expressions

**Figure 2:**
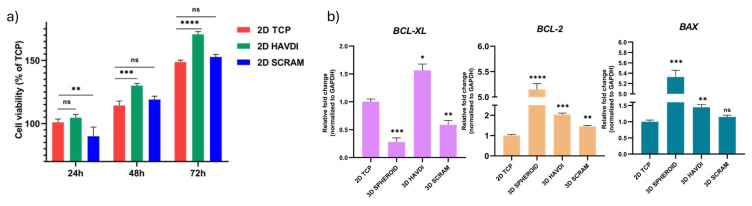
The effect of peptide amphiphile solutions on viability in 2D culture and on apoptosis-related genes in 3D culture. (a) The effect of peptides on the viability of ADMSCs in 2D cell culture, showing the percentage of viability rate. Data are expressed as mean ± SD with n = 6. (b) The fold changes in *BCL-XL*, *BCL-2*, and *BAX* gene expression in ADMSCs in response to peptide treatment in a 3D culture environment. ADMSCs cultured in 2D TCP served as the control group. Data are presented as mean ± SD with n = 3.

HAVDI and SCRAM peptide amphiphile solutions were tested in a 2D model to assess their effects on ADMSC metabolism and viability. After 24 h, the 2D HAVDI group showed 104% viability, while the 2D SCRAM group had 90% viability compared to the 2D TCP group (Figure 2a). At 48 h, the 2D TCP group exhibited 114% relative viability, whereas the 2D HAVDI and 2D SCRAM groups showed 130% and 119%, respectively, compared to the baseline level (Figure 1a). At 72 h, the 2D TCP group demonstrated 148% relative viability, while the 2D HAVDI and 2D SCRAM groups showed 170% and 152%, respectively, compared to the baseline. The 2D HAVDI group exhibited higher viability. The 3D environment was used to assess *BCL-XL*, *BCL-2*, and *BAX* expression. *BCL-2* genes play a crucial role in apoptosis (Kiraz et al., 2016). *BCL-XL* fold changes compared to the 2D TCP group were as follows: HAVDI: 1.5, SCRAM: 0.58, and Spheroid: 0.28. *BCL-2* expression fold changes compared to the 2D TCP group were 5.14, 2.03, and 1.45 for 3D Spheroid, HAVDI, and SCRAM. *BAX* fold changes were 5.32, 1.44, and 1.15 for 3D Spheroid, HAVDI, and SCRAM, respectively (Figure 2b). These findings indicate that the expression of BCL-XL and BAX was significantly upregulated in the HAVDI group compared to the 2D TCP group, suggesting an interplay between prosurvival and proapoptotic signals under the influence of the N-cadherin mimetic environment. Notably, BCL-2 expression also increased, albeit to a lesser extent, reflecting the complexity of apoptosis regulation in this 3D culture system.

### 3.4. N-cadherin biomimetic microenvironment impacts fibrosis by regulating tissue remodeling genes

**Figure 3:**
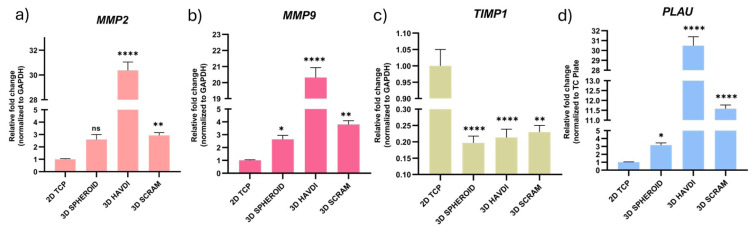
The effects of the N-cadherin biomimetic microenvironment on the expression of *MMP-2*, *MMP-9*, *TIMP1*, and *PLAU* genes in a 3D culture environment. The 2D TCP group was used as the control, and results were normalized to *GAPDH* expression. Only comparisons to the control group are presented. Data are expressed as mean ± SD with n = 3.

In the N-cadherin biomimetic microenvironment, the expression of extracellular matrix-regulating genes (*MMP-9*, *TIMP1*, *MMP-2*, *PLAU*) was examined. Genes involved in tissue remodeling, matrix breakdown, macrophage infiltration, and wound healing were chosen (Sautter et al., 2012). *MMP-2* gene expression fold changes compared to the 2D TCP group were 2.5, 30.3, and 2.9 for Spheroid, HAVDI, and SCRAM, respectively (Figure 3a). *MMP-9* gene expression fold increases were 2.6, 20.3, and 3.7 for 3D Spheroid, HAVDI, and SCRAM, respectively (Figure 3b). *TIMP1* gene expression fold changes were 0.19, 0.21, and 0.23 for 3D Spheroid, HAVDI, and SCRAM, respectively (Figure 3c). *PLAU* gene expression fold increases were 3.1, 30.4, and 11.5 for 3D Spheroid, HAVDI, and SCRAM, respectively (Figure 3d).

In the 3D HAVDI group, significant fold changes in extracellular matrix-regulating gene expression show that the N-cadherin biomimetic milieu may reduce fibrosis by influencing tissue remodeling. This suggests that the N-cadherin biomimetic environment may regulate wound healing and tissue repair by balancing synthesis and breakdown in ADMSCs’ extracellular matrix.

### 3.5. N-cadherin biomimetic microenvironment supports growth factor secretion of ADMSCs and upregulation of growth-factor-associated genes

**Figure 4:**
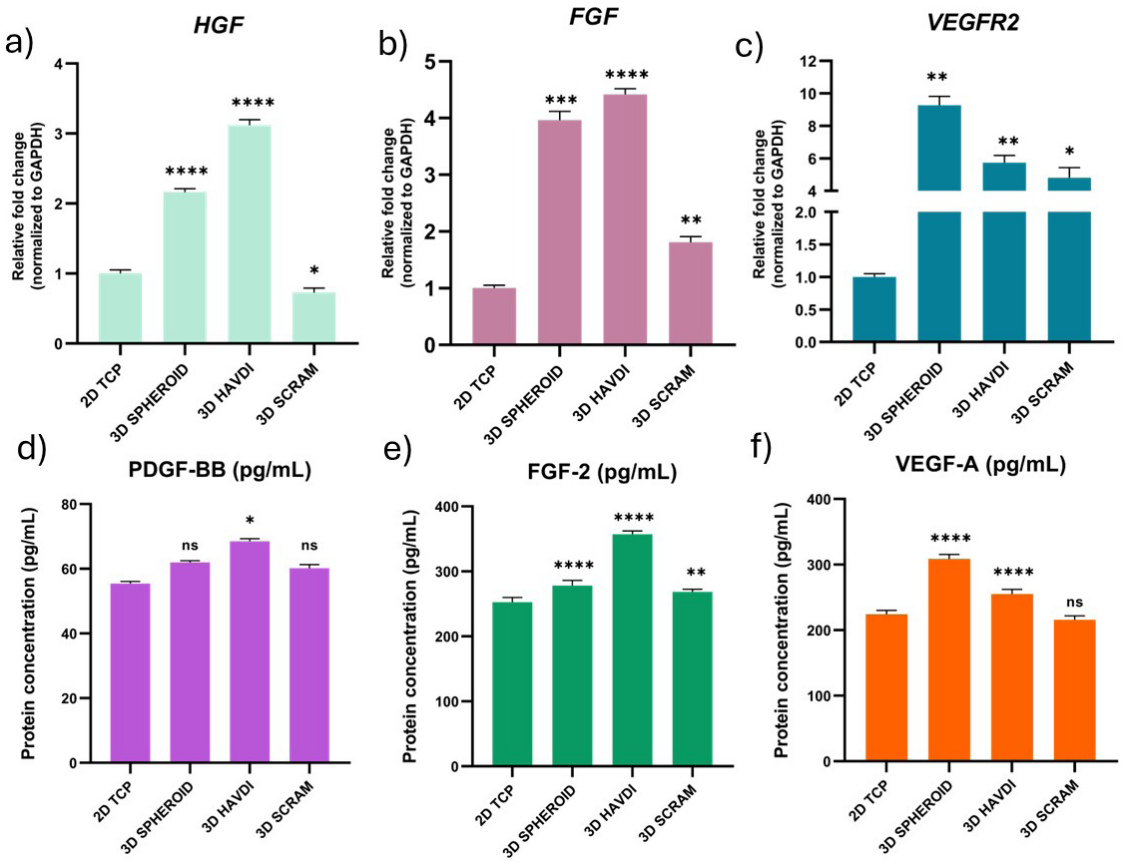
The effects of the N-cadherin biomimetic microenvironment on the fold change of genes associated with growth factors and protein levels in the supernatant. The impact of the N-cadherin biomimetic microenvironment on *HGF* (a), *FGF* (b), and *VEGFR2* (c) gene expression in the 2D TCP, 3D Spheroid, 3D HAVDI, and 3D SCRAM environments is shown. Additionally, the effects on PDGF-BB (d), FGF-2 (e), and VEGF-A (f) protein levels in the same environments are presented. ADMSCs cultured in 2D TCP were used as the control in qPCR experiments. Data are expressed as mean ± SD with n = 3.

The N-cadherin biomimetic milieu drastically altered *HGF*, *FGF*, and *VEGFR2* gene expression. Culture supernatant PDGF-BB, FGF-2, and VEGF-A levels were also tested. The environment’s VEGF protein levels regulate wound healing by promoting angiogenesis, wound closure, epidermal repair, and granulation tissue development (Johnson and Wilgus, 2014). FGF-2 also induces angiogenesis and enhances human wound healing (Farooq et al., 2021). In regeneration, PDGF-BB protein stabilizes newly created blood vessels (Caplan and Correa, 2011). The 3D Spheroid, 3D HAVDI, and 3D SCRAM groups had 2.1, 3.1, and 0.7 fold changes, respectively, in HGF gene expressions compared to the 2D TCP group (Figure 4a). In the 3D Spheroid, 3D HAVDI, and 3D SCRAM groups, *FGF* gene expressions changed by 3.9, 4.4, and 1.8, respectively, compared to the 2D TCP group (Figure 4b). The 3D Spheroid, 3D HAVDI, and 3D SCRAM groups had 9.2, 5.7, and 4.8 fold changes, relatively, for the VEGFR2 gene expression (Figure 4c). In addition, 2D TCP, 3D Spheroid, 3D HAVDI, and 3D SCRAM protein levels were examined. In particular, PDGF-BB protein levels were 55, 61, 68, and 60 pg/mL (Figure 4d). The levels of FGF-2 protein were 252, 278, 357, and 268 pg/mL (Figure 4d). Figure 4e shows VEGF-A protein levels of 224, 308, 255, and 215 pg/mL. The N-cadherin biomimetic microenvironment improves paracrine effects of ADMSCS and stimulates angiogenetic genes and growth factors in the 3D spheroid environment. The 3D HAVDI group showed high fold changes in gene expressions and growth factor protein secretion.

### 3.6. Conditioned media derived from the N-cadherin biomimetic microenvironment induces proliferation and migration of endothelial cells

**Figure 5:**
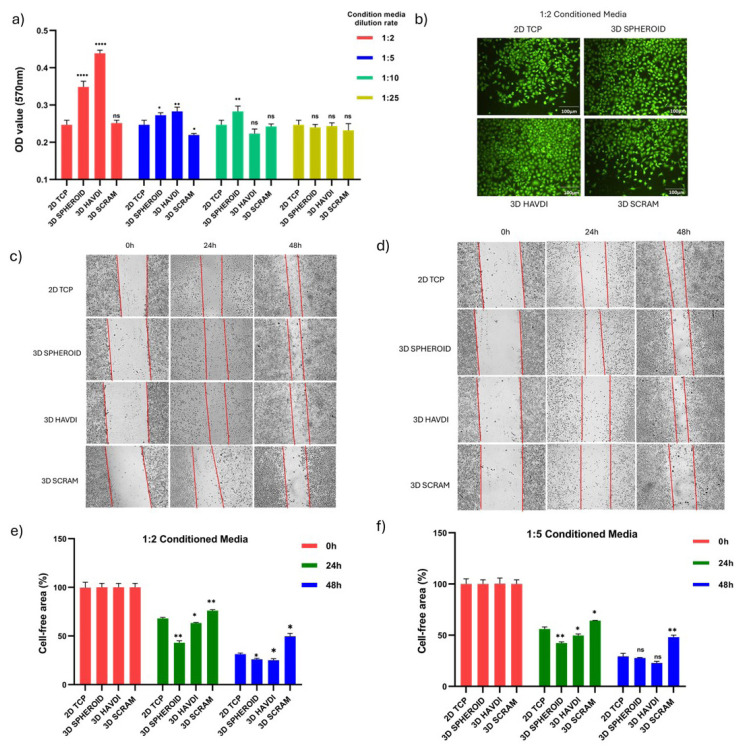
The effects of conditioned media obtained from ADMSCs cultured in an N-cadherin biomimetic microenvironment on the viability and migration of endothelial cells. (a) The 48-hour MTT results of conditioned media from 2D TCP, 3D Spheroid, 3D HAVDI, and 3D SCRAM cultures, diluted 2, 5, 10, and 25 times, on cell viability. Data are expressed as mean ± SD with n = 6. (b) Displays 48-h calcein staining at 10× magnification of endothelial cells cultured in 2-fold diluted conditioned media. Present migration images at 0, 24, and 48 h for endothelial cells treated with 2-fold and 5-fold diluted conditioned media (c,d), along with a % graph representing cell-free areas (e,f ). Data are expressed as mean ± SD with n = 6.

This section examines endothelial cell viability and migration in ADMSC-conditioned medium cultivated in an N-cadherin biomimetic environment. OD values from the MTT assay at 48 h were recorded for 2D TCP, 3D Spheroid, 3D HAVDI, and 3D SCRAM cultures diluted 2-, 5-, 10-, and 25-fold. In 2-fold diluted medium, OD values were 0.247 for 2D TCP, 0.348 for 3D Spheroid, 0.439 for 3D HAVDI, and 0.251 for 3D SCRAM. In 5-fold diluted medium, OD values were 0.248, 0.272, 0.282, and 0.219. In 10-fold diluted medium, OD values were 0.244, 0.282, 0.223, and 0.242. In 25-fold diluted medium, OD values were 0.249, 0.240, 0.243, and 0.232. The 3-D Spheroid and 3D HAVDI groups exhibited greater viability in 2-fold and 5-fold diluted medium (Figure 5a). In Figure 5b, 48-h calcein staining of cells in 2-fold diluted conditioned medium matches viability data. At 2-fold and 5-fold dilution rates, 3D HAVDI and 3D Spheroid conditioned media increased HUVEC cell viability.

Migration images (Figures 5c and 5d) and cell-free area percentage graphs (Figures 5e and 5f) were taken after endothelial cells were treated with 2-fold and 5-fold diluted conditioned media. HUVEC cells treated with 2-fold diluted conditioned media had 68% cell-free regions at 24 hours in the 2D TCP group, 43% in the 3D Spheroid group, 63% in the 3D HAVDI group, and 76% in the 3D SCRAM group. At 48 h, the cell-free areas were 49% for the 3D SCRAM group, 31% for the 2D TCP group, 26% for the 3D Spheroid group, and 25% for the 3D HAVDI group. At 24 hours, the 5-fold diluted 2D TCP group had 56% cell-free areas, the 3D Spheroid group 42%, the 3D HAVDI group 49%, and the 3D SCRAM group 64%. At 48 h, the cell-free areas were 29% for the 2D TCP group, 27% for the 3D Spheroid group, 22% for the 3D HAVDI group, and 48% for the 3D SCRAM group.

According to the cell-free area, HUVEC cells treated with conditioned media at 2-fold and 5-fold dilution rates from the 3D HAVDI and 3D Spheroid groups migrated faster than the 2D TCP group at 24 h. Only the 2-fold dilution group maintained this impact at 48 h.

## 4. Discussion

Recent studies have highlighted the promising role of N-cadherin HAVDI biomimetic sequences in enhancing the functionality of adipose-derived mesenchymal stem cells (ADMSCs) when incorporated into 1 mM hydrogels. These hydrogels have been shown to significantly increase ADMSC survival, reduce apoptosis, promote matrix remodeling, and enhance the expression of key growth factors and receptors, including *FGF*, *HGF*, and *VEGFR2*. Furthermore, the conditioned medium from ADMSCs has been found to boost the proliferation and migration of HUVECs, indicating a potential for improved angiogenesis.

ADMSCs are characterized by a specific surface marker profile, lacking CD14, CD34, CD45, and HLA-DR while expressing CD73, CD90, and CD105, which aligns with the criteria set by the International Society for Cellular Therapy (Nery et al., 2013). The transition to 3D culture systems has been shown to enhance the stemness, viability, and paracrine activities of MSCs, leading to improved angiogenic, antiinflammatory, and immunomodulatory properties compared to traditional 2D cultures (Zimmermann and Mcdevitt, 2014; Egger et al., 2018).

The HAVDI sequence has been linked to various cellular processes, including survival, adhesion, migration, and differentiation (Williams et al., 2002). Studies have demonstrated that hydrogels incorporating HAVDI can significantly enhance MSC proliferation and support their integration and movement (Cosgrove et al., 2016; Kaur and Roy 2021). The mechanism behind these effects includes the activation of β-catenin complexes, which promote proliferation through cyclin D1 and c-Myc gene expression, as well as the modulation of apoptosis pathways via N-cadherin interactions that inhibit proapoptotic signals (Wang et al., 2015).

Moreover, N-cadherin plays a crucial role in tissue remodeling and fibrosis, with its overexpression leading to the activation of genes associated with tissue remodeling, such as MMP10 and MMP13 (Yan et al., 2020; Ferrell et al., 2022). This suggests that N-cadherin could be a key player in mitigating fibrosis in conditions like ischemic heart disease (Yan et al., 2020).

The study also indicates that 3D cultures enhance the paracrine secretions of ADMSCs, with findings showing that secretomes from 3D cultures contain significantly higher levels of growth factors compared to 2D cultures. This enhanced secretion is believed to stimulate HUVEC migration and proliferation, further supporting the regenerative potential of ADMSCs in tissue engineering.

In conclusion, the findings underscore the biological advantages of N-cadherin biomimetic hydrogels in promoting ADMSC functionality and their potential applications in regenerative medicine. Future research is needed to explore the effects of varying concentrations of HAVDI hydrogels and their interactions with endothelial cells to fully understand their regenerative capabilities.

## 5. Conclusion

A three-dimensional N-cadherin-mimetic environment significantly enhances the regenerative and paracrine effects of MSCs compared to a 2D classical environment. Conditioned media from MSCs cultured in this 3D environment also enhances endothelial cell activation. Further research is necessary, particularly to assess the efficacy and safety of these peptide hydrogels in targeted clinical applications. Future studies should involve preclinical and clinical collaborations with tissue engineering researchers and clinicians to optimize 3D environments for enhancing MSCs’ therapeutic potential.

## Supplementary material

Figure S In vitro morphological and immunophenotypic characterization of ADMSCs. The ADMSCs displayed a fibroblast-like morphology under light microscopy (a), and their immunophenotypic characterization was confirmed through flow cytometry analysis (b).

## Figures and Tables

**Table:** Primers used for qRT-PCR expression analysis.

Gene name	Forward sequence	Reverse sequence
*GAPDH*	5′-GTCTCCTCTGACTTCAACAGCG-3′	5′-ACCACCCTGTTGCTGTAGCCAA-3′
*MMP-2*	5′-TTCATTTGGCGGACTGTGAC-3′	5′-GTGCTGGCTGAGTAGATCCA-3′
*MMP-9*	5′-GACGAGGGCCTGGAGTGT-3′	5′-TGTGCTGTAGGAAGCTCATCTC-3′
*BCL-2*	5′-GCCTTCTTTGAGTTCGGTGG-3′	5′-GAAATCAAACAGAGGCCGCA-3′
*BCL-XL*	5′-AAGAGAACAGGACTGAGGC-3′	5′-TTGCTTTACTGCTGCCATGG-3′
*BAX*	5′-CGCCCTTTTCTACTTTGCCA-3′	5′-CCAAAGTAGGAGAGGAGGCC-3′
*PLAU*	5′-GCCACACACTGCTTCATTGA-3′	5′-TATACATCGAGGGCAGGCAG-3′
*VEGFR2*	5′-ATCTGTGACTTTGGCTTGGC-3′	5′-TCCCACAGCAAAACACCAAA-3′
*TIMP-1*	5′-ACCCCTGGAGCACGGCT-3′	5′-CCCACCTTCCAAGTTAGTGACA-3′
*FGF*	5′-ATACGGCTCACAGACACCAA-3′	5′-TTCTGGCCATAGTGAGTCCG-3′
*HGF*	5′-CAGCTGGTATATGGCCCTG-3′	5′-TCAATCCAGTGTAGCCCCAG-3′

## Data Availability

Data available on request from the authors.
