# Supplementary figures and images for: N-cadherin mimetic hydrogels drive superior regenerative and paracrine responses in 3D cultures of adipose-derived mesenchymal stem cells
Source: Turk J Biol. 2025 Feb 3;49(2):209–18. doi: 10.55730/1300-0152.2738 (PMC12068664; doi:10.55730/1300-0152.2738)

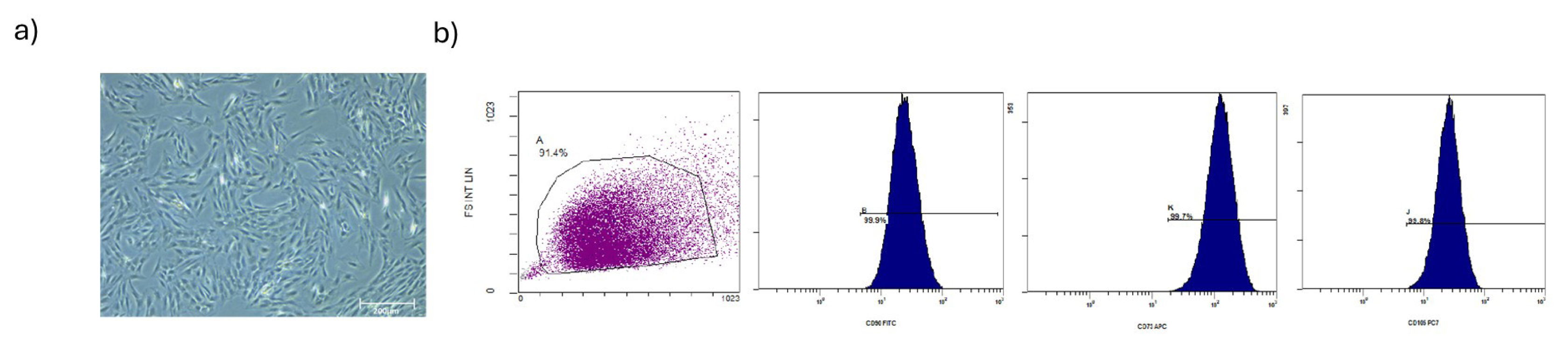

Supplement: Figure S — In vitro morphological and immunophenotypic characterization of ADMSCs. The ADMSCs displayed a fibroblast-like morphology under light microscopy (a), and their immunophenotypic characterization was confirmed through flow cytometry analysis (b). [file tjb-49-02-209s1.tif]
